# Regulation of ER stress-induced autophagy by GSK3*β*-TIP60-ULK1 pathway

**DOI:** 10.1038/cddis.2016.423

**Published:** 2016-12-29

**Authors:** Tiejian Nie, Shaosong Yang, Hongwei Ma, Lei Zhang, Fangfang Lu, Kai Tao, Ronglin Wang, Ruixin Yang, Lu Huang, Zixu Mao, Qian Yang

**Affiliations:** 1 Department of Neurosurgery, Tangdu Hospital, The Fourth Military Medical University, 569 Xinsi Road, Xi'an, Shaanxi 710038, China; 2 Department of Microbiology, Faculty of Preclinical Medicine, The Fourth Military Medical University, 169 Changlexi Road, Xi'an, Shaanxi 710032, China; 3 Department of Pharmacology and Neurology, Emory University School of Medicine, 615 Michael Street, Atlanta, GA 30322, USA

## Abstract

Endoplasmic reticulum (ER) stress is involved in many cellular processes. Emerging evidence suggests that ER stress can trigger autophagy; however, the mechanisms by which ER stress regulates autophagy and its role in this condition are not fully understood. HIV Tat-interactive protein, 60 kDa (TIP60) is a newly discovered acetyltransferase that can modulate autophagy flux by activating ULK1 upon growth factor deprivation. In this study, we investigated the mechanisms by which ER stress induces autophagy. We showed that ER stress activates glycogen synthase kinase-3*β* (GSK3*β*). This led to a GSK3*β*-dependent phosphorylation of TIP60, triggering a TIP60-mediated acetylation of ULK1 and activation of autophagy. Inhibition of either GSK3*β* or TIP60 acetylation activities significantly attenuated ER stress-induced autophagy. Moreover, enhancing the level of TIP60 attenuated the level of CHOP after ER stress, and reduced the ER stress-induced cell death. In contrast, expression of TIP60 mutant that could not be phosphorylated by GSK3*β* exacerbated the generation of CHOP and increased the ER stress-induced cell death. These findings reveal that ER stress engages the GSK3*β*-TIP60-ULK1 pathway to increase autophagy. Attenuation of this pathway renders cells more sensitive to and increases the toxicity of ER stress.

In eukaryotic cells, the key place for the canonical synthesis and maturation of proteins is the endoplasmic reticulum (ER). Under pathological stress conditions including hypoxia, energy deprivation, oxidative stress and imbalanced calcium levels, unfolded proteins will accumulate in the ER and disrupt ER homeostasis, thus leading to ER stress.^1^ ER stress is implicated in a wide range of diseases, including ischemia–reperfusion injury, diabetes and neurodegenerative diseases.^2, 3, 4, 5, 6^ To cope with the burden of unfolded proteins in its lumen, the ER activates intracellular signal-transduction pathways that are collectively termed the unfolded protein response (UPR).^7^ Emerging evidence indicate that ER stress can stimulate autophagy.^8, 9, 10, 11^ However, signal events involved in mediating ER stress-induced autophagy and the role that autophagy has in ER stress remain to be fully illustrated. Most studies in this area have focused on the upstream UPR pathways that may function to enhance autophagy flux under ER stress,^12, 13, 14, 15^ yet few details regarding the pathways downstream of ER to link this organelle to autophagic machinery have been identified.

Glycogen synthase kinase-3*β* (GSK3*β*) activity and ER stress are highly intertwined.^16, 17, 18, 19, 20, 21^ In addition, studies indicate that GSK3*β* also has a vital role in regulating autophagy at various levels under nutrient starvation.^22^ However, it is unclear that whether GSK3*β* participates in regulating ER stress-induced autophagy and what potential pathway may be required for this regulation. ULK1 is the mammalian counterpart of the yeast protein kinase Atg1, a key regulator of autophagy.^23, 24, 25^ Previous studies in yeast cells indicate that Atg1 kinase activity increases during ER stress,^8^ yet the signals and pathways that directly modulate ULK1 under ER stress remain to be clarified. A recent study has revealed that HIV Tat-interactive protein, 60 kDa (TIP60) directly acetylates and stimulates ULK1 to elicit autophagy under growth factor deprivation,^22^ yet it has not been tested whether this pathway functions in ER stress.

We show in this current study that ER stress can activate the GSK3*β*-TIP60-ULK1 pathway. Activation of this axis is essential in ER stress-induced autophagy as blocking this pathway markedly represses the activation of autophagy under ER stress. Furthermore, engaging this TIP60 pathway is critical for cells to maintain viability under ER stress as loss of this pathway increases ER-stress induced death. Thus, this study identifies GSK3*β*-TIP60-ULK1 as the key link that bridges ER stress and autophagy induction.

## Results

### ER stress induces TIP60 phosphorylation by activating GSK3*β*

**Figure 1:**
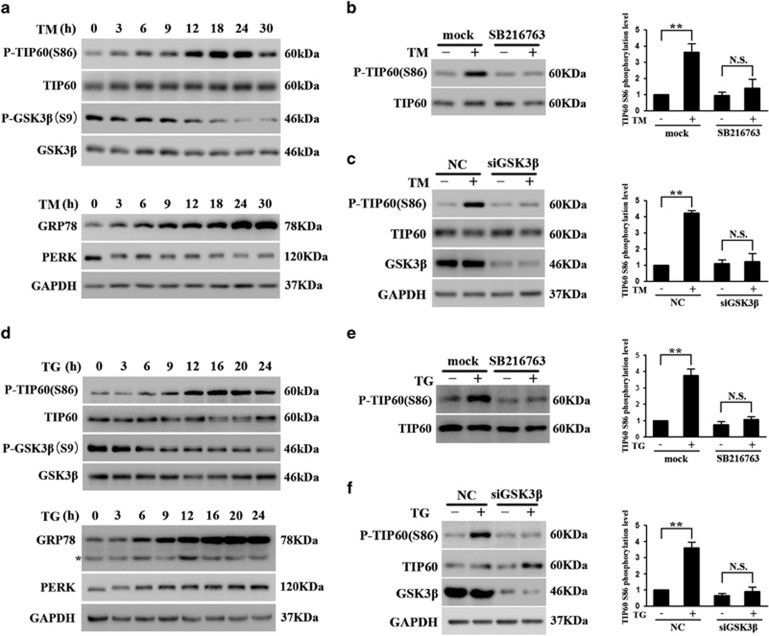
GSK3*β*-dependent phosphorylation of TIP60 under ER stress. (**a**) Time-dependent activation of GSK3*β* and TIP60 phosphorylation. HeLa cell lysates treated with TM (10 *μ*g/ml) induced a time-dependent increase in the level of GRP78 and PERK (bottom panel). The same lysates were blotted for total and phosphorylated TIP60 (S86) and GSK3*β* (Ser9) (top panel). (**b**) The effect of GSK3*β* inhibitor on TM-induced TIP60 phosphorylation. HeLa cells were co-treated with TM (10 *μ*g/ml) and DMSO (mock) or GSK3*β* inhibitor SB216763 (10 μM) for 24 h. The lysates were blotted for TIP60 as indicated. TIP60 Ser86 phosphorylation levels were calculated and normalized to total TIP60. Data represent the mean±S.E.M. of three independent experiments (**P<0.01; NS, not significant (two-way analysis of variance (ANOVA) followed by Tukey's test)). (**c**) The effect of GSK3*β* knockdown on TM-induced TIP60 phosphorylation. HeLa cells were transfected with control (NC) or GSK3*β* small interfering RNA (siRNA) for 48 h were treated with TM (10 *μ*g/ml) for 24 h. The endogenous GSK3*β* level (third panel) and total and phosphorylated TIP60 levels were determined. Data represent the mean±S.E.M. of three independent experiments (**P<0.01; NS, not significant (two-way ANOVA followed by Tukey's test)). (**d**–**f**) HeLa cells were treated with TG (1 *μ*M). The lysates were blotted as indicated. In (**e** and **f**), the detection of phosphorylated TIP60 (S86) was carried out as described in (**b** and **c**) after 16 h of TG exposure. Asterisk indicates the nonspecific band. Data represent the mean±S.E.M. of three independent experiments (**P<0.01; NS, not significant (two-way ANOVA followed by Tukey's test))

Tunicamycin (TM), the inhibitor of *N*-acetylglucosamine transferases, has been widely used to induce ER stress.^26^ We treated HeLa cells with TM and showed that exposure to TM leads to a time-dependent increase in the levels of ER stress markers – GRP78 and PERK,^27^ and causes PERK to migrate at slower rate, consistent with its phosphorylation and activation (Figure 1a), suggesting that TM effectively induces cellular ER stress. Using this cellular model, we tested the levels of GSK3*β* and TIP60. TM treatment induced a decrease in GSK3*β* phosphorylation at Ser9 and an increase in TIP60 phosphorylation at Ser86 in a time-dependent manner (Figure 1a).The TIP60 Ser86 phosphorylation level was clearly reduced when cells treated with a combination of TM and GSK3*β* inhibitor SB216763 (Figure 1b). To further confirm that GSK3*β* is responsible for TIP60 phosphorylation under ER stress, we knocked down endogenous GSK3*β* levels by transfecting cells with siRNAs specific for GSK3*β* and then treated cells with TM. Compared with the negative control siRNA, GSK3*β*-specific siRNAs reduced the level of GSK3*β* protein and completely abolished TM-induced TIP60 Ser86 phosphorylation (Figure 1c). Similar results were obtained when cells were treated with another ER stress inducer, thapsigargin (TG), the inhibitor of sarco/ER Ca^2+^ ATPase (SERCA),^28^ which further validated the GSK3*β*-TIP60 regulation pattern under ER stress (Figures 1d–f). Taken together, these findings indicate that ER stress can induce GSK3*β*-dependent TIP60 Ser86 phosphorylation.

### GSK3*β*-TIP60 axis regulates ULK1 acetylation and activation under ER stress

**Figure 2:**
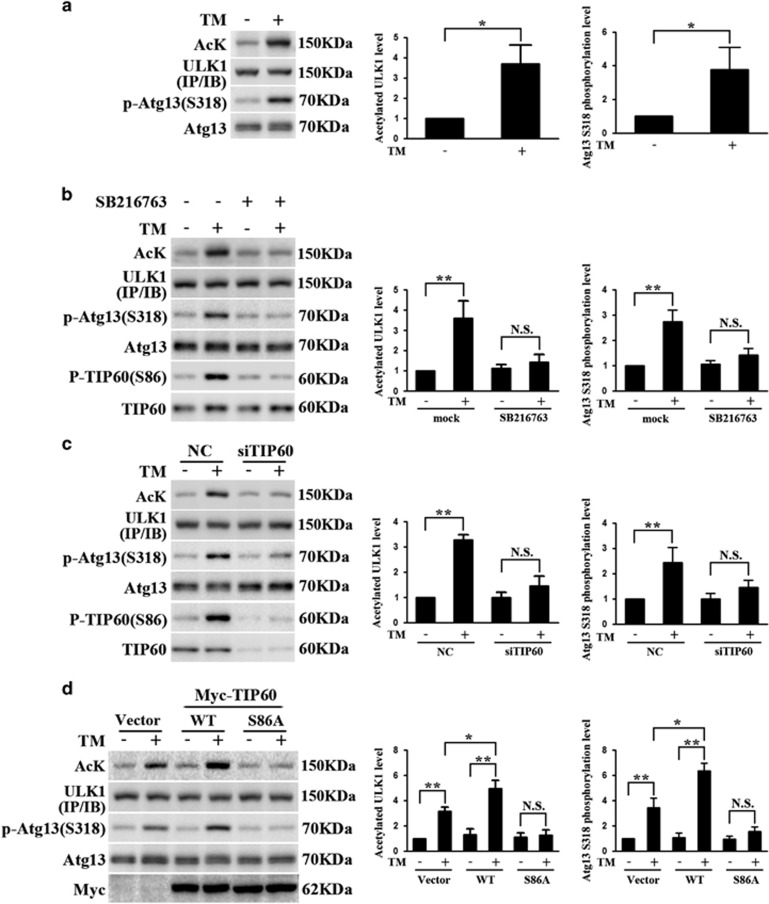

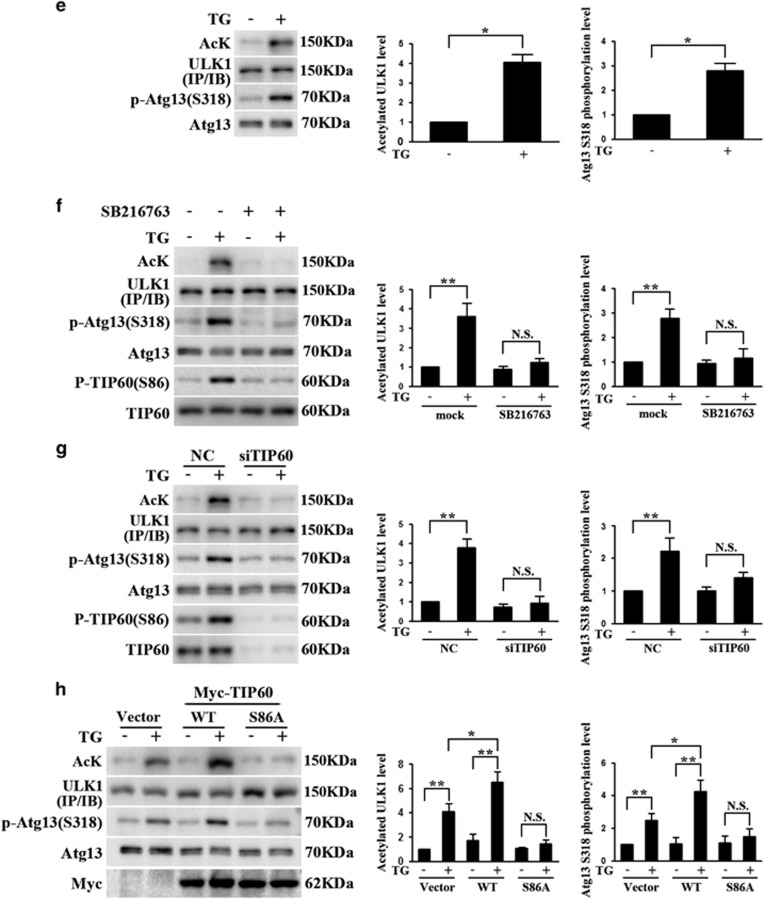
Acetylation of ULK1 by TIP60 under ER stress. (**a**) TM-induced acetylation of ULK1. Endogenous ULK1, which was immunoprecipitated from cell lysates that were harvested after 24 h TM (10 *μ*g/ml) treatment, was blotted with an antibody against acetylated lysine. The same membrane was reprobed with anti-ULK1 antibody. The total cell lysates was blotted with anti-P-Atg13 (S318) antibody and anti-Atg13 antibody. Ratio of acetylated ULK1 to total ULK1 and phosphorylated Atg13 to total Atg13 were calculated. Data represent the mean±S.E.M. of three independent experiments (*P<0.05 (Student's *t*-test)). (**b**) The effect of GSK3*β* inhibitor on TM-induced acetylation of ULK1. HeLa cells were treated as described in Figure 1b. The total and acetylated ULK1 levels were determined following immunoprecipitation as described in (**a**). Total cell lysates were blotted as indicated. Data represent the mean±S.E.M. of three independent experiments (**P<0.01; NS, not significant (two-way analysis of variance (ANOVA) followed by Tukey's test)). (c) The effect of TIP60 knockdown on TM-induced ULK1 acetylation. HeLa cells transfected with control (NC) or TIP60 small interfering RNA (siRNA) for 48 h were treated with TM (10 *μ*g/ml) for 24 h. The lysates were analyzed for total and acetylated ULK1. Total cell lysates were blotted as indicated. Data represent the mean±S.E.M. of three independent experiments (**P<0.01; NS, not significant (two-way ANOVA followed by Tukey's test)). (**d**) The effect of modulating TIP60 on TM-induced ULK1 acetylation. HeLa cells transfected with Vector, wild type (WT) or S86A TIP60 were treated with TM (10 *μ*g/ml) and analyzed for ULK1 acetylation after immunoprecipitation. Total cell lysates were blotted as indicated. Data represent the mean±S.E.M. of three independent experiments (*P<0.05; **P<0.01; NS, not significant (two-way ANOVA followed by Tukey's test)). (**e**–**h**) HeLa cells were treated with TG (1 μM). The lysates were blotted as indicated. The detection and analysis of acetylated ULK1, phosphorylated Atg13 (S318) and phosphorylated TIP60 (S86) were carried out as described in (**a**–**d**) after 16 h of TG exposure. Data represent the mean±S.E.M. of three independent experiments. (**e**) *P<0.05 (Student's *t* test); (**f**–**h**) *P<0.05; **P<0.01; NS, not significant (two-way ANOVA followed by Tukey's test)

To determine the role of GSK3*β* and TIP60 in ER stress-induced activation of autophagy, we first detected the acetylation of ULK1.The Atg13 Ser318 phosphorylation immunoblot was used to analyze the ULK1 kinase activity as reported before.^29^ Both TM and TG treatment increased the acetylation level of ULK1, a modification correlated with its activation as evidenced by increasing level of Atg13 Ser318 phosphorylation (Figures 2a and e). We then treated cells with a combination of ER stress inducers and GSK3*β* inhibitor SB216763, immunoprecipated ULK1 and analyzed its acetylation. The Atg13 Ser318 phosphorylation level was detected in the meantime. This analysis showed that SB216763 completely abolishes TM- or TG-induced ULK1 acetylation and activation (Figures 2b and f). Next, we transfected HeLa cells with siRNAs specific for TIP60 before TM or TG treatment, and performed similar experiment as described in Figure 2b. Our study revealed that TIP60 knockdown significantly attenuated TM- or TG-induced ULK1 acetylation (Figures 2c and g). In addition, overexpression of wild-type TIP60 further enhanced TM- or TG-induced ULK1 acetylation and activation; while increasing the level of TIP60 S86A mutant completely blocked ULK1 acetylation and activation in response to TM or TG treatment (Figures 2d and h). Thus, ER stress-induced ULK1 acetylation and activation requires GSK3*β* and TIP60.

### ER stress-induced activation of autophagy requires the GSK3*β*-TIP60-ULK1 signaling pathway

To corroborate with the ULK1 findings and testify whether GSK3*β* and TIP60 contribute to ER stress-induced autophagy, we tested the lipidation of microtubule-associated protein 1 light chain 3 (LC3) in response to ER stress. While TG is a robust inducer of ER stress, it also blocks the late stages of autophagy as the SERCA pump is also needed for fusion of autophagosomes with lysosomes.^30^ Thus, TM was mainly used here to carry out the autophagy assays.

**Figure 3:**
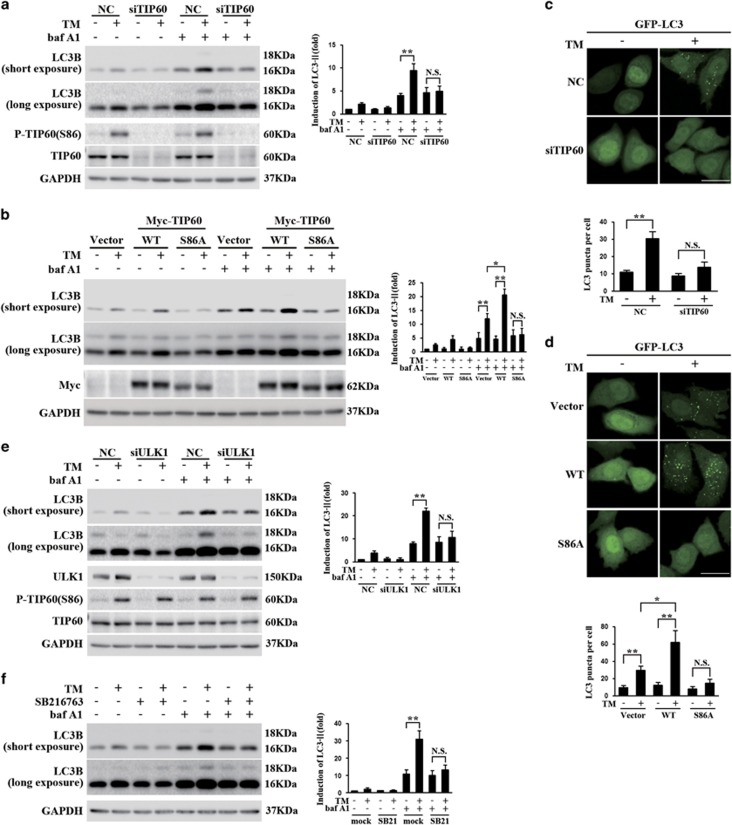
The essential role of GSK3*β*-TIP60-ULK1 pathway in ER stress-induced autophagy. (**a**) HeLa cells transfected with control (NC) or TIP60 small interfering RNA (siRNA) for 48 h were treated with TM (10 *μ*g/ml)and bafilomycin A1 (400 *μ*M) for 24 h. The lysates were blotted for LC3B, total and phosphorylated TIP60. The relative amounts of LC3BII were calculated from densitometry performed on immunoblots and normalized to the amount of glyceraldehyde 3-phosphate dehydrogenase (GAPDH). Data represent the mean±S.E.M. of three independent experiments (**P<0.01; NS, not significant (two-way analysis of variance (ANOVA) followed by Tukey's test)). (**b**) The effect of modulating TIP60 on TM-induced change in LC3BII. HeLa cells transfected with Vector, Myc-TIP60 (wild type (WT)) or Myc-TIP60 (S86A) for 20 h were treated with TM (10 *μ*g/ml) and bafilomycin A1 (400 *μ*M) for another 24 h. The lysates were immunoblotted as indicated. Data represent the mean±S.E.M. of three independent experiments (*P<0.05; **P<0.01; NS, not significant (two-way ANOVA followed by Tukey's test)). (**c**) The effect of knockdown TIP60 on TM-induced LC3B puncta formation. HeLa cells co-transfected with GFP-LC3 (green) and TIP60 small interfering RNA (siRNA) for 48 h were treated with TM (10 *μ*g/ml) for 24 h and scored for the number of puncta. Quantification shown above represents the mean GFP puncta per cell (*n*=12) from three independent experiments±S.E.M. (**P<0.01; NS, not significant (two-way ANOVA followed by Tukey's test); scale bar, 20 *μ*m). (**d**) The effect of modulating TIP60 on TM-induced LC3B puncta formation. HeLa cells were transfected as indicated and then treated with TM (10 *μ*g/ml) for another 24 h. LC3B puncta formation was evaluated as described in (**c**) (*P<0.05; **P<0.01, NS, not significant (two-way ANOVA followed by Tukey's test); scale bar, 20 *μ*m). (**e**) The effect of ULK1 knockdown on TM-induced change in LC3BII. HeLa cells were transfected with control (NC) or ULK1 siRNA for 48 h were treated with TM (10 *μ*g/ml) and bafilomycin A1 (400 μM) for another 24 h. The lysates were immunoblotted as indicated. Data represent the mean±S.E.M. of three independent experiments (**P<0.01; NS, not significant (two-way ANOVA followed by Tukey's test)). (**f**) The effect of GSK3*β* inhibitor on TM-induced LC3BII change. HeLa cells were treated with TM (10 *μ*g/ml) with or without SB216763 (10 *μ*M) and bafilomycin A1 (400 *μ*M) for 24 h. LC3BII level was determined. Data represent the mean±S.E.M. of three independent experiments (**P<0.01; NS, not significant (two-way ANOVA followed by Tukey's test))

Cells were transfected with TIP60-specific siRNAs and then treated with TM. As autophagy is a dynamic process, bafilomycin A1, a vacuolar H^+^-ATPase inhibitor, was used to inhibit lysosomal events to differentiate changes in autophagosome formation *versus* autophagosome degradation.^31^ TM treatment with control siRNA increased LC3BII. In contrast, TIP60 knockdown greatly attenuated the effect of TM on LC3BII induction (Figure 3a). Using a similar approach as described in Figure 2d, we showed that increasing the level of WT TIP60 further enhanced the induction of LC3BII following TM treatment. However, TIP60 S86A mutant greatly reduced the induction of LC3BII by TM (Figure 3b). To strength these immunoblot findings, we transfected cells with a GFP-LC3B and analyzed the effects of modulating TIP60 on the GFP-LC3B vesicles formation following TM treatment by fluorescence microscope. Consistent with the previous western blot results, TM treatment resulted in an increase in GFP-LC3 puncta formation, suggesting autophagy induction.^32^ TIP60 knockdown markedly inhibited TM-induced GFP-LC3 puncta formation compared with the control group (Figure 3c). Similar to the findings in Figure 2d,WT TIP60 overexpression caused more GFP-LC3 puncta formation in comparison with the Vector group and TIP60 S86A overexpression significantly reduced the level of GFP-LC3 puncta formation compared with WT TIP60 group (Figure 3d). To assess whether this process in dependent on ULK1 activation, we carried TM treatment after transfecting cells with ULK1-specific siRNAs. The LC3BII production was greatly decreased after ULK1 knockdown, while the level of TIP60 and its Ser86 phosphorylation was not altered (Figure 3e). Taken together, these findings indicate that TM-induced changes in autophagy require TIP60, the TIP60 Ser86 phosphorylation and subsequent ULK1 activation.

We then treated cells with SB216763 and showed that GSK3*β* inhibition greatly repressed autophagy induction under TM treatment (Figure 3f). This finding indicated that GSK3*β* is an important sensor kinase that participates in autophagy induction during ER stress by triggering a specific signaling axis.

### Oxidative stress causes ER stress and activates the GSK3*β*-TIP60-ULK1 signaling pathway

**Figure 4:**
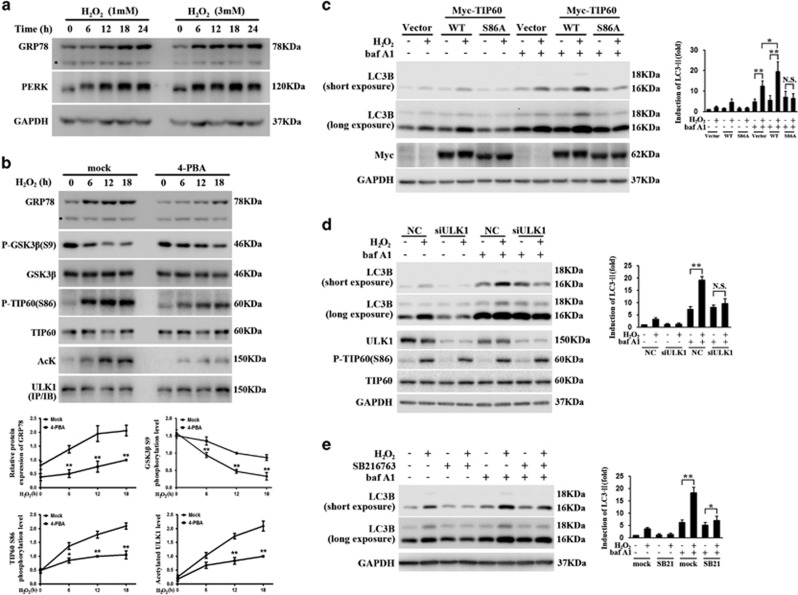
Hydrogen peroxide (H_2_O_2_)-induced ER stress and activation of GSK3*β*-TIP60-ULK1 pathway. (**a**) H_2_O_2_-induced ER stress. HeLa cells were treated with H_2_O_2_ (1 or 3 mM). Cell lysates were gathered at the indicated time points and blotted for the ER stress markers as indicated. (**b**) H_2_O_2_-induced activation of GSK3*β*, TIP60 and ULK1. HeLa cells were treated with H_2_O_2_ (1 mM) with or without 4-PBA (2 mM) for the indicated time. Cell lysates were blotted as indicated. Densitometric analyses of the western blots are shown as curves. Data represent mean±S.E.M. of three independent experiments (*P<0.05; **P<0.01 (two-tailed Student's *t*-test)). (**c**–**e**) The effect of modulating GSK3*β*-TIP60-ULK1 pathway on H_2_O_2_-induced change in LC3BII. HeLa cells were treated as described in Figures 3b, e and f, except that H_2_O_2_ (1 mM)and bafilomycin A1 (400 *μ*M) for another 18 h. The lysates were immunoblotted as indicated. LC3BII level was determined. Data represent the mean±S.E.M. of three independent experiments (*P<0.05; **P<0.01; NS, not significant (two-way analysis of variance (ANOVA) followed by Tukey's test))

To strengthen the findings made with ER stress inducers – TM and TG – we examined the role of GSK3*β*-TIP60-ULK1 axis in autophagy response provoked by another ER stress inducer. Many studies have reported that H_2_O_2_, a common inducer of oxidative stress, can also effectively evoke ER stress.^32, 33, 34, 35^ Therefore, we treated HeLa cells with different concentrations of H_2_O_2_ and showed that H_2_O_2_ causes a dose- and time-dependent increase in GRP78 and a mobility shift of PERK (Figure 4a), demonstrating the induction of ER stress.

Exposing cells to H_2_O_2_ significantly activated GSK3*β* evidenced by its gradual dephosphorylation, led to increased phosphorylation of TIP60 and enhanced ULK1 acetylation (Figure 4b). To confirm that these changes are indeed mediated by ER stress, we co-treated cells with H_2_O_2_ and 4-phenylbutyrate acid (4-PBA), a well-known chemical chaperone used widely to specifically attenuate ER stress.^36^ The results showed that inhibition of ER stress by 4-PBA largely reversed the effects of H_2_O_2_ on the activation of GSK3*β*, phosphorylation of TIP60 and acetylation of ULK1 (Figure 4b). We then conducted the autophagy assays. Similar to TM treatment, modulating the activity of TIP60 by WT TIP60 or TIP60 S86A overexpression caused opposite effects in LC3BII production (Figure 4c), and the role of TIP60 in autophagy is also ULK1-dependent as evidenced by ULK1 knockdown (Figure 4d). Moreover, suppressing the GSK3*β* activity by SB216763 exhibited the inhibitory effects in H_2_O_2_-induced autophagy induction (Figure 4e). Thus, different types of ER stressors engage GSK3*β*-TIP60-ULK1 pathway to regulate autophagy induction.

### ER stress-induced TIP60 activation modulates the UPR

**Figure 5:**
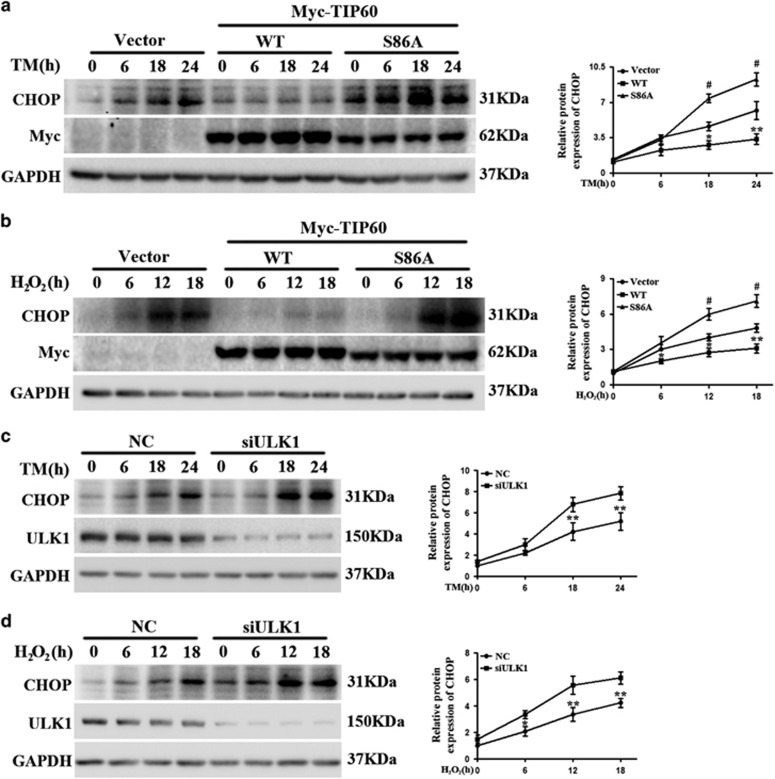
The role of TIP60 and ULK1 in ER stress-induced changes in C/EBP Homologous Protein (CHOP). (**a** and **b**) The effect of modulating TIP60 on TM- or hydrogen peroxide (H_2_O_2_)-induced changes in CHOP levels. HeLa cells were transfected as indicated for 20 h and then treated with TM (10 *μ*g/ml) or H_2_O_2_ (1 mM). Cell lysates were blotted for CHOP, Myc and glyceraldehyde 3-phosphate dehydrogenase (GAPDH). The right curves show the quantification of relative levels of CHOP. Data represent the mean±S.E.M. of three independent experiments (*P<0.05; **P<0.01 versus Vector; ^#^*P*<0.01 versus wild type (WT) (two-way analysis of variance (ANOVA) followed by Tukey's test)). (**c** and **d**) The effect of ULK1 knockdown on TM- or H_2_O_2_-induced changes in CHOP levels. HeLa cells were transfected with control (NC) or ULK1 small interfering RNA (siRNA) for 48 h were treated with TM (10 *μ*g/ml) or H_2_O_2_ (1 mM). CHOP level was determined. Data represent the mean±S.E.M. of three independent experiments (*P<0.05; **P<0.01 (two-tailed Student's *t*-test))

Prolonged ER stress is accompanied by a sustained UPR, and this is known to induce apoptosis-related proteins including the persistent activation of the PERK-ATF4-CHOP pathway,^37, 38^ and comprise cellular viability. To evaluate the role of GSK3*β*-TIP60-ULK1 pathway on CHOP generation, we treated cells with TM after transfecting the cells with Vector, WT TIP60 or TIP60 S86A and determined the levels of CHOP at different time points. Western blot analysis revealed that prolonged TM or H_2_O_2_ treatment causes an increase in CHOP production. WT TIP60 substantially attenuated TM-induced increase in CHOP while TIP60 S86A greatly exacerbated their induction by TM or H_2_O_2_ in a time-dependent manner (Figures 5a and b). Similar results were obtained when TM or H_2_O_2_ exposures were carried out after ULK1 depletion (Figures 5c and d). Taken together, these results suggest that ER stress-induced activation of the GSK3*β*-TIP60-ULK1 may be a key cellular mechanism that modulates the UPR and restrains CHOP generation to maintain cellular homeostasis.

### GSK3*β*-TIP60-ULK1 pathway is essential for promoting cell survival and limiting cell death under ER stress

**Figure 6:**
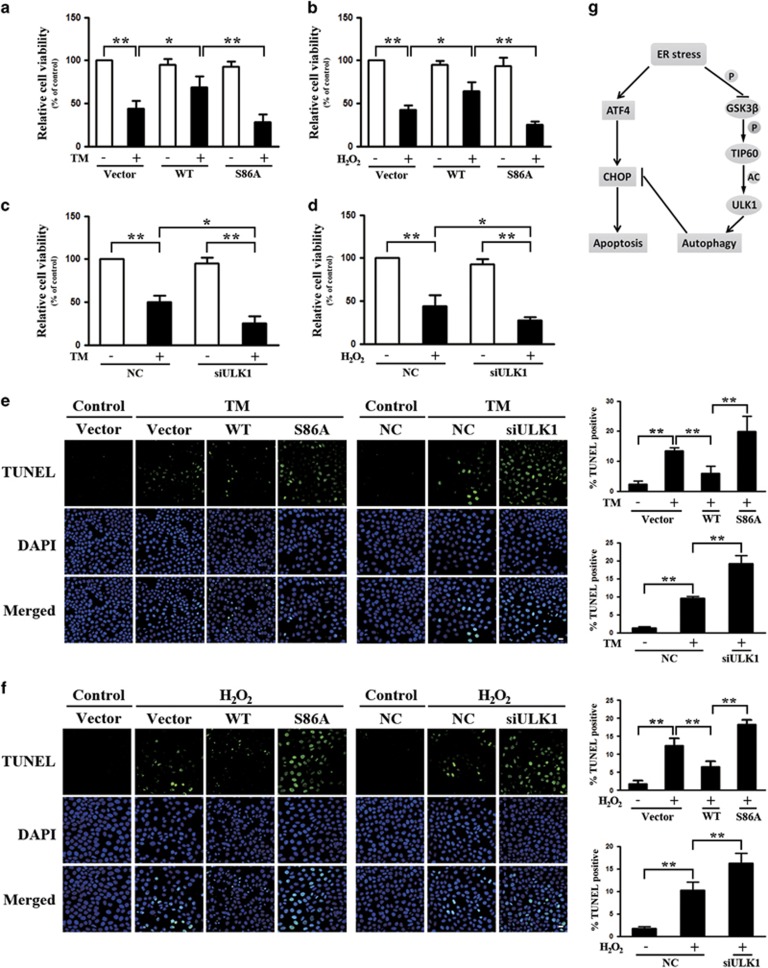
The role of TIP60 and ULK1 in ER stress-induced cellular apoptosis. (**a**, **c** and **e**) The effect of modulating TIP60 and ULK1 on TM-induced changes in HeLa cell viability. HeLa cells were transfected with Vector, Myc-TIP60 (wild type (WT)) or Myc-TIP60 (S86A) for 20 h or transfected with control (NC) or ULK1 small interfering RNA (siRNA) for 48 h and then treated with TM (10 *μ*g/ml)for another 24 h. Cell viability was analyzed with MTT (**a** and **c**), or imaged with TUNEL assay (scale bar=20 *μ*m). (**e**) Data represent the mean±S.E.M. of three independent experiments (*P<0.05; **P<0.01 (two-way analysis of variance (ANOVA) followed by Tukey's test)). (**b**, **d** and **f**) The effect of modulating TIP60 and ULK1 on hydrogen peroxide (H_2_O_2_)-induced changes in HeLa cell viability. HeLa cells were transfected as indicated and then treated with H_2_O_2_ (1 mM) for 18 h. The measurement of MTT and TUNEL assay were carried out as described above in (**a**, **c** and **e**) (scale bar=20 *μ*m). Data represent the mean±S.E.M. of three independent experiments (**P<0.01 (two-way ANOVA followed by Tukey's test)). (**g**) Model for the role of GSK3*β*-TIP60-ULK1 pathway in autophagy induction and cell survival under ER stress

Because the GSK3*β*-TIP60-ULK1 pathway can regulate CHOP generation, we investigated its role in cell survival using both TM and H_2_O_2_ models. We transfected cells with Vector, WT TIP60 or TIP60 S86A, exposed cells to either TM or H_2_O_2_ and measured cellular viability by using the MTT (3-(4,5-dimethylthiazol-2-yl)-2,5-diphenyltetrazolium bromide) assay. Prolonged treatment of cells with either TM or H_2_O_2_ led to significant cell death (Figures 6a–d). WT TIP60 overexpression strongly enhanced cell viability compared with the Vector, whereas overexpression of TIP60 S86A caused more cellular death (Figures 6a and b). We conducted ULK1 inhibition studies in parallel by transfecting cells with ULK1-specific siRNAs. ULK1 depletion also exacerbated the TM- or H_2_O_2_-induced cell death(Figures 6c and d). These findings were consistent with TUNEL assay results (Figures 6e and f). Therefore, the level of TIP60 activity modulates cellular sensitivity to ER stress-induced toxicity and this role of TIP60 is autophagy-dependent.

## Discussion

It is well known that ER stress triggers UPR to initiate cellular attempt of restoring its homeostasis.^39^ Autophagy is a conserved cellular catabolic mechanism for degrading and recycling cytosolic, long-lived or aggregated proteins and excess or defective organelles, thus promoting the cell survival response to nutrient starvation and stress conditions.^40, 41^ Mounting evidence indicates that autophagy is induced by ER stress in organisms from yeast to mammals. However, the role of autophagy in ER stress is somewhat controversial or dynamic and the signaling mechanisms linking ER stress to autophagy remain not fully delineated. On the one hand, ER-induced autophagy may act as a protective mechanism to backup the ER-associated degradation (ERAD)^8, 42, 43^ pathway in helping handle the superfluous cell burden under ER stress. On the other hand, it can initiate programmed cell death if ER stress cannot be relieved.^10, 44, 45^ Our current investigation is the first to demonstrate that the GSK3*β*-TIP60 axis, at least in part, accounts for ER stress-induced activation of autophagy. This involves ULK1 activation. We found that TM or H_2_O_2_ treatment induces TIP60 phosphorylation via GSK3*β*. Activated TIP60 then acetylates ULK1 to promote autophagy. Interfering with this pathway by inhibiting either GSK3*β* or TIP60 function can substantially attenuate the ER stress-induced increase in autophagy. Increase in TIP60 activity further enhances ER stress-induced autophagy. Thus, the GSK3*β*-TIP60 signal appears to be both necessary and sufficient to modulate ER stress-induced autophagy.

GSK3*β* is a serine-threonine kinase that is involved in a wide range of cellular processes important for the cellular homeostasis of energy and growth.^46, 47^ Previous studies have demonstrated that GSK3*β* is activated under ER stress,^19, 20, 21^ yet none of these studies has examined the relationship between GSK3*β* and ER stress-induced autophagy. Our study indicates that GSK3*β* participates in ER stress-induced autophagy by activating the TIP60-ULK1 pathway. GSK3*β* inhibition partially prevented the autophagy induction under ER stress. Therefore, it is clear from our study that GSK3*β* is necessary to sense ER stress and engage autophagic machinery in eukaryote cells. Taken together with previous reports,^22^ it indicates that GSK3*β* is required for both ER stress-dependent and -independent induction of autophagy. The role of GSK3*β* in cellular viability upon ER stress is complex. One study reported that GSK3*β* phosphorylates p53 and inhibits p53-mediated cell apoptosis under ER stress.^17^ Other studies reported that GSK3 inhibition represses caspase-3 activation and subsequent cell death induced by ER stress.^16^ Our data show that GSK3*β* activation promotes autophagy initiation following ER stress, which allows cells to recover homeostasis and is clearly beneficial for cells. Therefore, the exact role of this kinase in ER stress is complex and may, in part, depend on cellular context and the distinct targets and signaling pathways engaged by GSK3*β*.

To elucidate the potential effect of this autophagy pathway, we explored the relationship between the UPR and the GSK3*β*-TIP60-ULK1 axis.The UPR primarily consists of three distinct ER stress transducers: IRE1, ATF6 and PERK.^7^ It is an adaptive response that increases the ER folding capacity; however, it can also induce apoptosis if ER stress cannot be alleviated.^48, 49^ Activated PERK can phosphorylate eIF2*α* to reduce the load of newly synthesized proteins through translation initiation inhibition. In the meantime, phosphorylated eIF2*α* induces the expression of a transcription factor – ATF4.^7^ ATF4 promotes the generation of proteins that help restore homeostasis in the ER.^50^ ATF4 also upregulates the apoptosis-inducing protein – CHOP. Although CHOP is a short-lived protein, prolonged and strong ER stress parallels with persistent CHOP expression, thus leading to cell apoptosis.^37, 38, 51^ Our data reveal that the activity of GSK3*β*-TIP60-ULK1 pathway correlates closely with CHOP and with cellular viability, and the higher the pathway activity is, the lower the CHOP level is. Notably, the modulating effect of this pathway on CHOP level appears to be most pronounced at the later time point during ER stress, consistent with the possibility that the dynamic change of autophagy during the late stage of prolonged ER stress may be part of the critical signal that exerts significant impact on cellular ability to commit to apoptotic path.

In summary, ER stress inducers such as TM or H_2_O_2_ activate the GSK3*β*-TIP60-ULK1 pathway to induce autophagy. Losing this pathway renders cells more vulnerable to TM- or H_2_O_2_-induced cell stress and triggers higher level of apoptosis. Conversely, strengthening this pathway confers resistance to ER stress-induced apoptosis. The exact molecular targets by which autophagy relieves ER stress remain to be fully identified. Given the well-known function of autophagy, it is possible that enhanced autophagy flux may, in parallel to ERAD, help lessen the ER burden by degrading the misfolded protein and damaged organelles from various subcellular sources.

## Materials and methods

### Plasmids, antibodies and chemicals

The Myc-TIP60 (WT) and Myc-TIP60 (S86A) constructs were gifts from Dr. Sheng-Cai Lin (State Key Laboratory of Cellular Stress Biology, School of Life Sciences, Xiamen University, Fujian, China). The GFP-LC3B construct was purchased from Invitrogen (Carlsbad, CA, USA). The following antibodies were used: anti-phospho-TIP60 (Ser86) antibody (a gift from Dr. Xiaotong Li, State Key Laboratory of Cellular Stress Biology, School of Life Sciences, Xiamen University, Fujian, China); anti-TIP60 (ab14584), anti-GRP78 (ab21685), anti-Atg13 (ab201467) antibodies (Abcam, Cambridge, MA, USA); and anti-GSK3*β* (no. 12456), anti-phospho-GSK3*β* (Ser9) (no. 5558), anti-GAPDH (no. 5174), anti-PERK (no. 3192), anti-ULK1 (no. 8054), anti-LC3B (no. 3868), anti-c-Myc (no. 2276), anti-acetylated-lysine (no. 9441), anti-CHOP (no. 2895) antibodies (Cell Signalling Technology, Beverly, MA, USA); anti-phospho-Atg13 (Ser318) (PAB19948) antibody (Abnova, Taipei, Taiwan). TG (T9033), TM (T7765), SB216763 (S3442) and 4-PBA (SML0309) were purchased from Sigma, St. Louis, MO, USA and bafilomycin A1 (ab120497) from Abcam.

### Cell culture and plasmid transfection

HeLa cells were maintained in Dulbecco's modified Eagle's medium supplemented with 10% fetal bovine serum (Gibco, Grand Island, NY, USA), 2 mM L-glutamine, 100 IU penicillin and 100 mg/ml streptomycin at 37 °C in a humidified incubator with 5% CO_2_. Cells were transfected with expression plasmids using the Lipofectamine 2000 reagent (Invitrogen) according to the manufacturer's instructions.

### RNA interference

The homo-GSK3*β*, TIP60 and ULK1 siRNAs were purchased from GenePharma (Shanghai, China). The siRNA specific for GSK3*β* was: 5′-GUCGCCAUCAAGAAAGUAUTT-3′ for TIP60 was: 5′-CCACAGGAACUCACCACAUTT-3′ for ULK1 was: 5′-UCACUGACCUGCUCCUUAA-3′. A non-targeting siRNA was used as a control with sense (5′-UUCUCCGAACGUGUCACGUTT-3′) and antisense (5′-ACGUGACACGUUCGGAGAATT-3′). Cells were transfectedwith siRNAs using the Lipofectamine 2000 reagent according to the manufacturer's instructions.

### Immunoblot analysis

Cells were washed three times with ice-cold PBS, scraped in cold PBS, collected by centrifugation at 800 × *g* and then incubated in ice-cold lysis buffer (150 mM NaCl, 50 mM Tris (pH 7.5), 1 mM EDTA, 1 mM EGTA, 2 mM DTT, 1% Triton X-100, a protease inhibitor cocktail (Roche, St. Louis, MO, USA) and a phosphatase inhibitor cocktail (Roche)) for 30 min. Samples were then clarified by centrifugation and the protein content was measured using a BCA Protein Assay Kit (Thermo Scientific, Rockford, IL, USA) according to the manufacturer's instructions. Equal amounts of protein were separated on 10–15% polyacrylamide gels, transferred onto PVDF membranes (Millipore, Billerica, MA, USA) and subjected to western blotting using the standard protocol, and visualized using the chemiluminescent detection system (ECL; Bio-Rad, Hercules, CA, USA). Densitometry quantitation of the bands was performed using the ImageJ software (National Institutes of Health, Bethesda, MD, USA).

### Immunoprecipitation

Cell lysates were harvested and protein content was measured as described above. The lysates containing equal amounts of protein were immunoprecipitated with anti-ULK1 antibody and protein A/G plus-agarose immunoprecipitation reagent (SantaCruz Biotechnology, Dallas, TX, USA) overnight at 4 °C. Thereafter, the precipitants were washed five times with ice-cold lysis buffer, and the immunocomplexes were eluted with sample buffer containing 1% SDS for10 min at 100 °C and analyzed by 10% SDS–PAGE using the antiacetylated lysine and anti-ULK1 antibodies.

### MTT assay

Cells were seeded in 96-well plates at a proper density. MTT (5 mg/ml) (Sigma) was added to each well after the indicated treatment and the plate was incubated for another 4 h. One hundred and fifty microliters of dimethylsulfoxide (DMSO) (Sigma) was added to each well after the supernatant was removed. The plate was mixed thoroughly for 10 min and then the optical density (OD) value was measured at 490 nm.

### TUNEL assay

TUNEL assay was performed using the FragEL DNA Fragmentation Detection Kit, Fluorescent (Millipore). HeLa cells were seeded onto poly-lysine-coated chamber slides, transfected and treated as indicated. Then, the cells were fixed with 4% paraformaldehyde for 20 min, permeabilized with 0.1% Triton X-100 on ice for 2 min and incubated with TUNEL reaction mixture at 37 °C for 1 h according to the manufacturer's protocol. The cells were then stained with DAPI (Roche) and detected under a laser scanning confocal microscope (Olympus, Olympus Canada, Richmond Hill, ON, Canada).

### Statistical analysis

All data were expressed as the mean±S.E.M. from at least three independent experiments. One- or two-way ANOVA followed by Tukey's post-test was used to compare values among different experimental groups using the SPSS Statistics 19.0 program. Student's *t*-test and two-tailed Student's *t*-test were used for experiments that contain only two groups. A value of *P*<0.05 was considered statistically significant, and a value of *P*<0.01 was considered highly significant.
